# The Influence of PSA Pre-Anodization of AA2024 on PEO Coating Formation: Composition, Microstructure, Corrosion, and Wear Behaviors

**DOI:** 10.3390/ma11122428

**Published:** 2018-11-30

**Authors:** Maria Serdechnova, Sergey A. Karpushenkov, Larisa S. Karpushenkava, Maksim Starykevich, Mario G. S. Ferreira, Theodor Hack, Mariia H. Iuzviuk, Igor A. Zobkalo, Carsten Blawert, Mikhail L. Zheludkevich

**Affiliations:** 1 Institute of Materials Research, Helmholtz-Zentrum Geesthacht, Max-Planck-Straβe 1, 21502 Geesthacht, Germany; carsten.blawert@hzg.de (C.B.); mikhail.zheludkevich@hzg.de (M.L.Z.); 2 Faculty of Chemistry, Belarusian State University, Nezavisimosti Avenue 4, 220030 Minsk, Belarus; ksazaslavl@mail.ru (S.A.K.); karpushenkava@bsu.by (L.S.K.); 3 Department of Materials and Ceramic Engineering/CICECO—Aveiro Institute of Materials, University of Aveiro, 3810-193 Aveiro, Portugal; mstarykevich@ua.pt (M.S.); mgferreira@ua.pt (M.G.S.F.); 4 Airbus Group Innovations, 81663 Munich, Germany; theo.hack@airbus.com; 5 B. P. Konstantinov Petersburg Nuclear Physics Institute, NRC Kurchatov Institute, Orlova Roshcha 1, 188300 Gatchina, Russia; qesada21@gmail.com (M.H.I.); Zobkalo_IA@pnpi.nrcki.ru (I.A.Z.); 6 Institute for Materials Science, Faculty of Engineering, University of Kiel, Kaiserstrasse 2, 24143 Kiel, Germany

**Keywords:** AA2024, plasma electrolytic oxidation, phosphoric-sulfuric acid anodizing, corrosion resistance, wear resistance, post-treatment

## Abstract

In the frame of the current work, it was shown that plasma electrolytic oxidation (PEO) treatment can be applied on top of phosphoric sulfuric acid (PSA) anodized aluminum alloy AA2024. Being hard and well-adherent to the substrate, PEO layers improve both corrosion and wear resistance of the material. To facilitate PEO formation and achieve a dense layer, the systematic analysis of PEO layer formation on the preliminary PSA anodized layer was performed in this work. The microstructure, morphology, and composition of formed PEO coatings were investigated using scanning electron microscopy (SEM), x-ray diffraction (XRD), and glow-discharge optical emission spectroscopy (GDOES). It was shown that under constant current treatment conditions, the PSA layer survived under the applied voltage of 350 V, whilst 400 V was an intermediate stage; and under 450 V, the PSA layer was fully converted after 5 min of the treatment. The comparison test with PEO formation on the bare material was performed. It was confirmed that during the “sparking” mode (400 V) of PEO formation, the PEO coatings, formed on PSA treated AA2024, were more wear resistant than the same PEO coatings on bare AA2024.

## 1. Introduction

Aluminum alloy AA2024 is widely used, specifically, in the aerospace industry due to its superior mechanical properties [1,2]. However, the corrosion susceptibility of this alloy is high because of the microgalvanic coupling between the alloy matrix and the present intermetallics, especially those containing Cu. The main solutions currently implemented in the aeronautical industry include the formation of high barrier layers together with active protection components based on corrosion inhibitors present in different parts of the protective system. Historically the pre-treatments, anodic layers, and corrosion protection primers were based on Cr(VI) technologies. However, many countries have banned the use of protective systems containing toxic chromates [3]. Following this, a lot of research has focused on the formation of corrosion protection systems that fit with the environmental regulations [4,5].

Phosphoric sulfuric acid (PSA) anodizing is nowadays widely used in the aeronautical industries to prevent toxic chromium containing compounds [6,7]. During this anodization, a thin anodic oxide layer with a thickness of about 1 to 3 μm is formed, which is intended to improve the adhesion between the metallic substrate and the applied top-coat. However, in some places, the surface of the aluminum alloy is subject to enhanced mechanical properties and resistance to wear in particular. Those regions should be additionally treated to prevent mechanical damage and to extend the lifetime of the materials in use. Local engineering for only local reinforcement of components provides an additional design option, if the general properties are not sufficient.

The application of plasma electrolytic oxidation (PEO) treatment to aluminum alloys leads to a significant increase of the alloys hardness, as well as providing improved wear and corrosion resistance due to the formation of ceramic-like coatings [8,9,10,11,12], as a result of short-living spark discharges under high voltage conditions in alkaline environmentally friendly electrolytes. The most used electrolytes for PEO treatment of aluminum alloys are composed of mixtures of several components, e.g., sodium silicate (0.5–50 g L^−1^), potassium or sodium hydroxide (1–50 g L^−1^), sodium aluminate (2–20 g L^−1^), sodium or potassium carbonate (up to 500 g L^−1^), sodium fluoride (0.5–20 g L^−1^), and hexametaphosphate (up to 150 g L^−1^) [13]. Silicate and/or silicate-phosphate electrolytes are the most environmentally friendly electrolytes, when they do not contain fluorides and/or heavy metal salt additives [14,15,16,17,18]. In this work, we investigated whether the properties of a PSA anodized layer can be improved with a subsequent PEO treatment. We also checked whether this might be an option for the local reinforcement of a PSA layer. A comparative study of the structure, morphology, and properties of such PEO coatings with and without anodizing pre-treatment was performed.

## 2. Experimental

### 2.1. Materials

The following chemicals were used for surface preparation, PSA synthesis, and PEO synthesis: sodium metasilicate (Na_2_SiO_3_, 44–47% SiO_2_, Sigma-Aldrich Chemie GmbH, Darmstadt, Germany), sodium hydroxide (NaOH, >99%, Merck KGaA, Darmstadt, Germany), sodium dihydrogen phosphate (Na_2_H_2_P_2_O_7_, 98%, Chempur, Karlsruhe, Germany), nitric acid (HNO_3_, 65%, Merck KGaA, Darmstadt, Germany), sulfuric acid (H_2_SO_4_, 95–97%, Merck KGaA, Darmstadt, Germany), ortho-phosphoric acid (H_3_PO_4_, 87 % extra pure, Merck KGaA, Darmstadt, Germany), and sodium chloride (NaCl, 99.98%, Fisher Chemical, Loughborough, UK). Deionized water was used as a solvent. АА2024-T3 with a nominal composition of [wt.%]: 3.8–4.9 Cu, 0.5 Fe, 0.1 Cr, 1.2–1.8 Mg, 0.3–0.9 Mn, 0.5 Si, 0.15 Ti, 0.25 Zn, 0.15 others, and Al balance—was used (specimen dimensions of 20 × 30 × 2 mm^3^).

### 2.2. Surface Treatment

Before the PSA anodizing procedure, aluminum specimens were degreased and etched according to the standard commercial procedure (alkaline cleaning in Metaclean T2001 (Chemie Vertrieb Hannover, Hannover, Germany) at 65 °C for 15 min, alkaline etching in P3 Almeco (Turco Chemie, Hamburg, Germany) at 35 °C for 3 min, acid etching in Turco Liquid Smutgo NC (Turco Chemie, Hamburg, Germany)) at 40 °C for 5 min each, followed by rinsing in deionized water and drying under air.

The anodizing process was performed in a phosphoric acid/sulfuric acid bath at 27 °C and carried out at 18 V for 23 min following the method described in Reference [19]. After the anodizing step, the specimens were rinsed with deionized water and dried under air conditions.

Prior to the PEO treatment on bare AA2024, the surface of the aluminum alloy was chemically etched according to the procedure outlined in References [20,21,22]. Briefly, AA2024 (without PSA treatment) was firstly degreased in a mixture of ethyl alcohol and acetone (1:1), etched in 20% NaOH for 60 s, then rinsed with deionized water, then it was additionally desmutted in 65% HNO_3_ for 60 s. Finally, the AA2024 was rinsed with deionized water and dried under compressed air. PSA treated samples did not undergo any pretreatments before PEO application.

PEO processing was carried out using a pulsed DC power supply with a duty cycle of t_on_:t_off_ = 1 ms:9 ms and maximum average current density was limited to 5 A dm^−2^. The aqueous electrolyte, comprising of 9 g L^−1^ Na_2_SiO_3_, 2 g L^−1^ NaOH and 11 g L^−1^ Na_2_H_2_P_2_O_7_, was continuously stirred during the treatment and kept at 20 ± 2 °C using a water cooling system. The counter-electrode was made of stainless steel. After PEO treatment, the specimens were rinsed with deionized water and dried under compressed air. Three different voltages (350, 400, and 450 V) and three different times (5, 15, and 30 min) were applied to the PEO processing.

### 2.3. Characterization Methods

Surface morphology and cross-sections of the formed coatings were examined using a Tescan Vega3 SB scanning electron microscope (SEM, Brno-Kohoutovice, Czech Republic). Cross-sections were prepared by grinding through successive grades of silicon carbide (SiC) paper, with final polishing done to a 1 μm diamond finish.

Phase compositions of the samples were characterized using a Bruker D8 Advance diffractometer (Karlsruhe, Germany, Ni-filtered Cu Kα radiation (1.5406 Å), step size 0.02°, dwell time ~1.5 s) at room temperature with a glancing angle of 0.5, 1, 2, and 3°.

Glow discharge optical emission spectroscopy (GDOES) analysis of the coatings was done using a HORIBA GD-Profiler 2 with a copper anode of 4 mm in diameter (Horiba, Longjumeau, France). Argon sputtering of the sample surface occurred at a pressure of 650 Pa and power of 30 W. A minimum of four measurements were performed on each sample.

The dry sliding wear behavior of the PEO coatings was assessed with an oscillating ball-on-disc tribometer (Tribotec AB, Mölnlycke, Sweden), with an AISI 52100 steel ball of 6 mm diameter as the static friction counterpart (RGPBALLS S.r.l., Cinisello Balsamo, Italy). The wear tests were performed at ambient conditions (25 ± 2 °C and 36–44% relative humidity) with different loads ranging from 1 to 10 N and on an oscillating amplitude of 10 mm with a sliding velocity of 5 mm s^−1^, and a total sliding distance of 12,000 mm. Arithmetic mean surface roughness (R_a_) usually called average roughness of the coatings was measured using a Hommel profilometer T1000 (Hommelwerke GmbH, S-Schwenningen, Germany).

Electrochemical impedance spectroscopy (EIS) measurements were conducted (three electrode cell) in a stirred aqueous 0.5 wt.% NaCl solution at 22 ± 0.5 °C using a computer controlled potentiostat system (Gamry interface 1000: Gamry instruments, Warminster, PA, USA). All potentials were measured with respect to the Ag/AgCl reference electrode. A platinum mesh was used as the counter electrode. The measurements were performed at different times up to 24 h via applying a sinusoidal perturbation of 10 mV RMS amplitude and a frequency sweep from 30 kHz to 0.01 Hz, using a working electrode area of 0.5 cm^2^. All measurements were repeated twice with good reproducibility. The impedance spectra were analyzed with the Gamry Echem Analyst software (Gamry instruments, Warminster, PA, USA). The errors for the individual parameters of the equivalent electrical circuits (such as CPE and R) are presented in the results discussion.

## 3. Results and Discussion

### 3.1. Voltage Evolution and Current Density Variation

**Figure 1:**
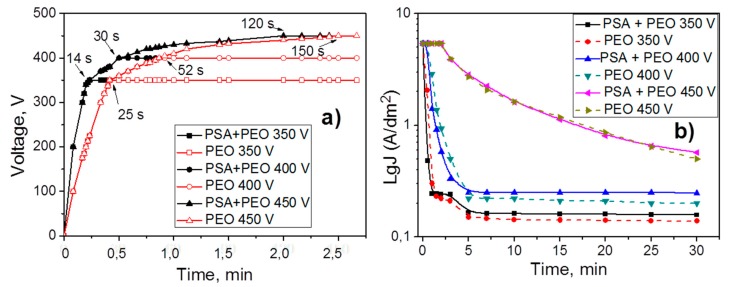
Voltage evolution (**a**) and current density (**b**) plots as a function of the plasma electrolytic oxidation (PEO) processing time for 350, 400, and 450 V without and with phosphoric sulfuric acid (PSA) pre-anodizing.

Figure 1 shows the voltage and current density as a function of the PEO processing time of aluminum alloy AA2024 without and with PSA pre-treatment. Different stages of PEO treatment can be distinguished in the graphs. During the first stage (until the voltage reaches the predetermined values of 350, 400, and 450 V, respectively), the current density was constant at 5 A dm^−2^ (so called galvanostatic or constant current oxidation (CC-mode)). During the second period, the process proceeded at a constant voltage (potentiostatic or constant voltage oxidation (CV-mode)) and the current density changed. At the moment of the CC to CV mode transition, the current density decreased immediately.

The samples which were originally covered with a PSA layer reached the defined voltage faster compared to the samples without the PSA treatment (the transition between CC and CV modes in Figure 1 requires, 14 and 25 s at 350 V, 30 and 52 s at 400 V, and 120 and 150 s at 450 V, respectively). It could be attributed to better dielectric properties of the existing PSA layer on the surface. As a result, PEO processing on the PSA coated aluminum alloy required a higher voltage in order to reach the same current densities as was observed for the bare samples.

Under the conditions of the so-called “soft sparking” [23,24] (relatively low voltage (350 and 400 V), the CC to CV transition happened much earlier (not later than after 52 s). Thus, the exposure period to high current density was significantly shorter at the lower voltages. Afterwards, the current density decreased very sharply to the minimum values within 3–5 min of the PEO treatment and then it remained constant. Thus, during this time, the PSA layer could not be fully converted into a PEO coating leading to differences in the current density of the PSA pre-anodized samples and the bare AA2024. For the PSA pre-anodized samples, sparking started earlier on the surface of the alloy and the current density decreased faster, since the PSA layer acted as a pre-formed passive oxide film. However, the higher final current densities of the pre-anodized specimens indicated that they were more defective than those, which were directly grown on the AA2024.

It was observed that the nature of sparking was also changed under different applied voltages. During oxidation processes under 350 V, sparking was hardly visually observed (only in the dark could a glow at 350 V on a metal substrate be seen). It could be explained that the sparks were very small and visually indistinguishable. At 400 V, the number of sparks decreased, but the size did not change, and at 450 V, the number of sparks decreased, but at the same time the sparks were significantly enlarged.

### 3.2. Surface and Cross-Sectional Morphologies

The SEM investigation of the surface morphology of PEO covered AA2024 (350 and 400 V) showed that under a voltage of 350 V, PSA + PEO coatings had more round pores with an average diameter of 0.1–1 μm, and in the case of PEO coatings only, the pores became elongated with a width of 0.5–1 μm and a length up to 5 μm. It was also observed that under a voltage of 400 V, the size of the large round pores in the PSA + PEO coatings reached 2 μm, and for the PEO coatings, pores of a more oval shape up to 2–3 μm were visible. At a voltage of 450 V, a sharp surface relief with pores of irregular shape and dimensions of up to 5 μm was observed for both cases. No difference in surface morphology for the PEO and PSA + PEO coatings was observed at voltages of 450 V, since at this voltage the PSA layer practically did not influence the formation of the PEO coatings.

**Figure 2:**
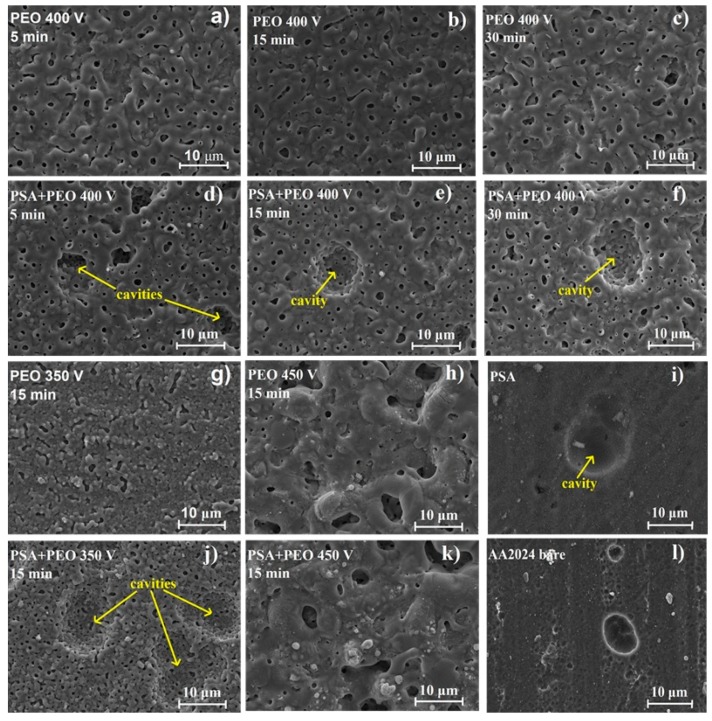
Scanning electron microscopy (SEM) images of the coating surfaces of bare AA2024, PSA coated AA2024, PEO coated AA2024, and PEO coated AA2024 with PSA pre-treatments after different voltages and times of PEO processing (**a**) PEO 400 V 5 min, (**b**) PEO 400 V 15 min, (**c**) PEO 400 V 30 min, (**d**) PSA + PEO 400 V 5 min, (**e**) PSA + PEO 400 V 15 min, (**f**) PSA + PEO 400 V 30 min, (**g**) PEO 350 V 15 min, (**h**) PEO 450 V 15 min, (**i**) PSA, (**j**) PSA + PEO 350 V 15 min, (**k**) PSA + PEO 450 V 15 min, (**l**) AA2024 bare.

**Figure 3:**
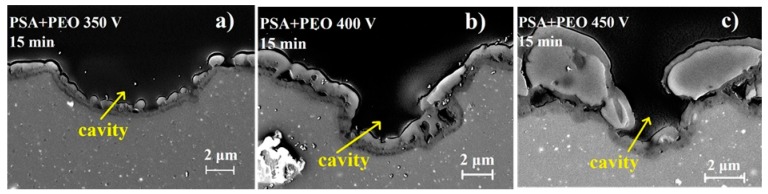
SEM images of the coatings cross-sections for PEO coated AA2024 with PSA pre-treatments and pits of etching after PEO processing at different voltages (**a**) PSA + PEO 350 V 15 min, (**b**) PSA + PEO 400 V 15 min, (**c**) PSA + PEO 450 V 30 min.

As can be seen in Figure 2, the surface of the aluminum after etching contains cavities, which were formed due to the removal of intermetallics that were part of the initial alloy. These cavities were clearly distinguishable on the uncoated alloy, on the PSA-treated surfaces, and on the PSA + PEO coatings with a small thickness 0.5–2.5 µm (350–400 V, 5–30 min). The presence of cavities on the samples with a PSA layer in comparison with only PEO treated samples was related to the more aggressive conditions of AA20204 surface preparation (etching) prior to PSA anodizing [19]. The coating relief repeated all the changes in the relief of the original surface without the formation of additional defects (as can be seen in Figure 2d–f surface view, and Figure 3 cross-section view).

**Figure 4:**
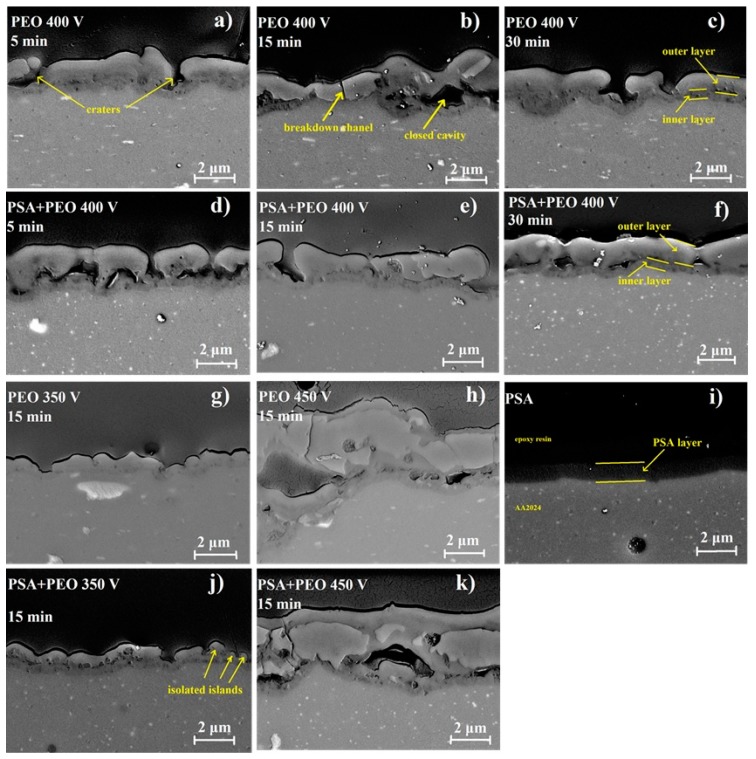
SEM images of the coatings cross-sections for bare AA2024, PSA coated AA2024, PEO coated AA2024, and PEO coated AA2024 with PSA pre-treatments after different voltages and times of PEO processing (**a**) PEO 400 V 5 min, (**b**) PEO 400 V 15 min, (**c**) PEO 400 V 30 min, (**d**) PSA + PEO 400 V 5 min, (**e**) PSA + PEO 400 V 15 min, (**f**) PSA + PEO 400 V 30 min, (**g**) PEO 350 V 15 min, (**h**) PEO 450 V 15 min, (**i**) PSA, (**j**) PSA + PEO 350 V 15 min, (**k**) PSA + PEO 450 V 15 min.

From the cross-sections presented in Figure 4, the PEO coatings do not have uniform thickness, i.e., the coatings consist of a thicker outer porous layer and a thin inner layer, as was also confirmed in other works [25,26].

The cross-sections of PEO coatings (Figure 3 and Figure 4) revealed that for all PEO coatings (350–450 V), a characteristic dense inner layer existed near the interface with a thickness of about 0.5 μm, which did not depend on the applied voltage. However, the thickness of the outer layer and its defectiveness strongly depended on the oxidation conditions. At 350 V for PEO and PSA + PEO coatings, the outer layer did not fully cover the surface; and the isolated islands of PEO could be distinguished. Internal closed cavities of about 100 nm for PEO coatings and 100–300 nm for PSA + PEO coatings were observed. At 400 V, breakdown channels and craters of up to 1 μm in size were visible in the outer layer of the PEO coatings, as well as large cavities between the inner and outer layers of the coating with a width of up to 1 μm and a length of up to 2 μm. In the case of PSA + PEO coatings, a denser structure of the outer layer was visible, and the cavity size did not exceed 1 μm. At a voltage of 450 V, the above-mentioned defects and internal cavities with a width of up to 2 μm and a length of up to 5 μm were observed in both cases. All the thicknesses were measured 10 times at different positions of the sample and the average results were presented.

**Figure 5:**
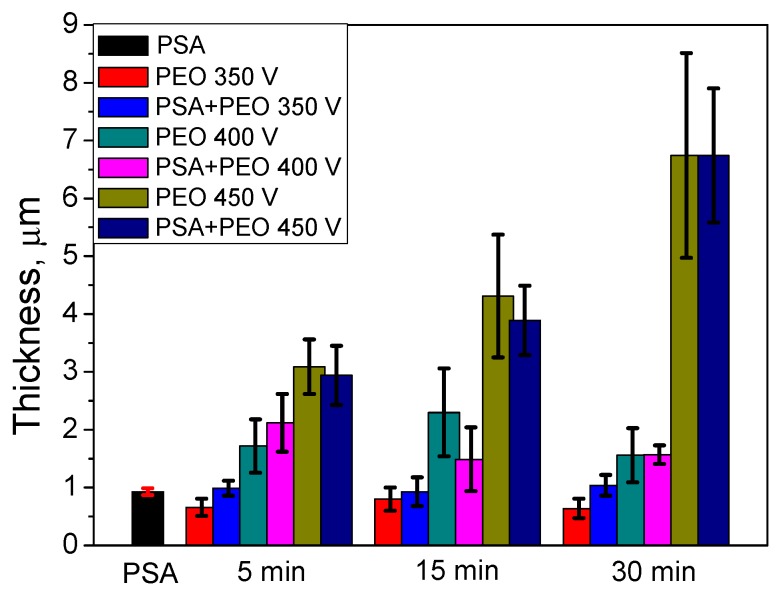
The thickness measured from the cross-section of PEO coated AA2024 at different voltages (350–450 V) and times (5–30 min) without and with PSA pre-treatments.

Using cross-section SEM analysis (Figure 4), the average thickness of the coatings was determined. The preliminary formed PSA layer influenced not only the parameters of the PEO coating formation, but also the coating thickness. Under a voltage of 350 V, the PSA pre-treatment increased the thickness, whilst under higher voltages, the behavior was the opposite and the PEO coatings that formed on the bare material were thicker (Figure 3 and Figure 5). It could be explained that under 350 and 400 V, the PSA layer was not completely converted to a PEO layer and the resulting total thickness of the PSA + PEO coatings was higher than the PEO coatings on bare alloy. The thinner layer of PEO + PSA under 450 V in comparison with PEO on bare AA2024 could be explained by the complete transformation of the anodic PSA layer, which reduced the total time available for PEO formation.

### 3.3. Roughness of the PEO Coating

Under lower voltages of PEO processing, the formation of coatings with relatively uniform structure (both on bare and on PSA pre-treated AA2024 alloy) is observed (Figure 4). However, with the increase in the oxidation voltage, the larger cavities, located between the dense inner barrier layer and the outer looser layer of the PEO coating, were noticed for the PEO coatings obtained after PSA anodizing. The tendency of pore number reduction, as well as enlargement of the individual pore diameter, as a function of voltage as described previously in Reference [27] was observed, leading to the increase in roughness of the layer.

**Figure 6:**
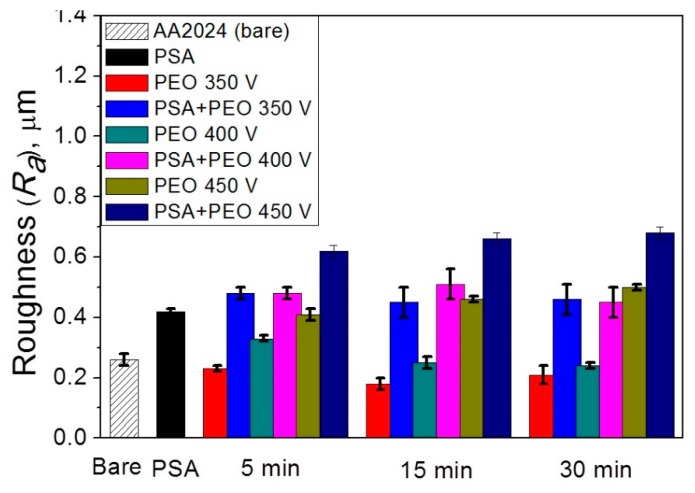
The roughness R_a_ (b) of the PSA and the PEO coating, at different voltages (350–450 V) and times (5–30 min) without and with PSA pre-treatments.

It can be seen from Figure 6 that the initial AA2024 alloy and the alloy coated with the PSA layer have different roughness (0.26 ± 0.02 and 0.42 μm ± 0.01, respectively), which also affects the roughness of the PEO coatings, especially those formed at voltages of 350 and 400 V. Therefore, for the PEO coatings on the bare AA2024 and with the PSA layer obtained at 350 V, the roughness was 0.18–0.23 ± 0.03 μm and 0.45–0.48 ± 0.05 μm, respectively. The PEO coatings formed under more stringent oxidation conditions at a voltage of 450 V had significantly higher roughness values, i.e., 0.62–0.68 ± 0.02 μm vs 0.41–0.50 ± 0.02 μm for PEO coatings on the initial alloy and on a previously applied PSA layer, respectively.

### 3.4. Chemical and Phase Composition of the Coatings

**Figure 7:**
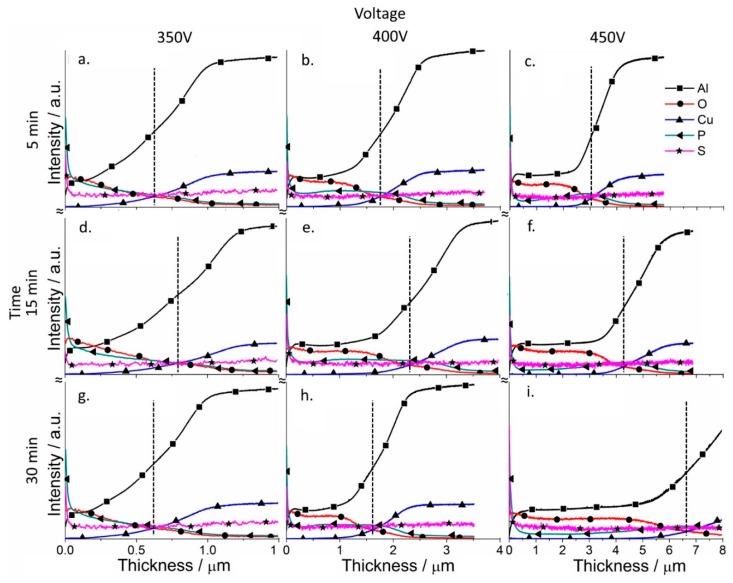
Qualitative depth profile measured by GDOES of PEO coated AA2024 at different voltages (350–450 V (vertical)) and times (5–30 min (horizontal)), without PSA pre-treatments (the dashed line indicates the thickness of the coatings).

**Figure 8:**
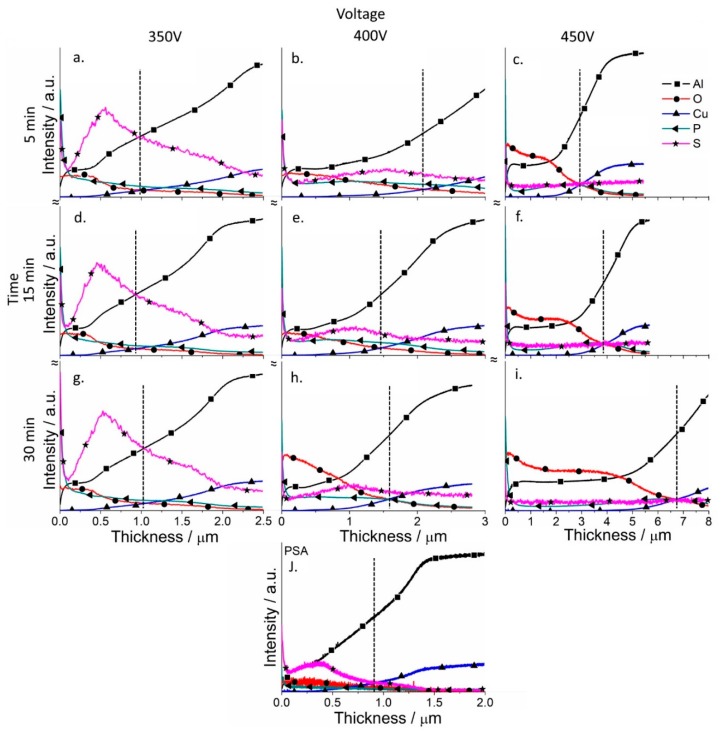
Qualitative depth profile measured by GDOES of PSA (**j**) and PSA-PEO (**a**–**i**) coated AA2024 at different voltages (350–450 V (vertical)) and times (5–30 min (horizontal)), with PSA pre-treatments (the dashed line indicates the thickness of the coatings).

Depth profile analysis for the elements of the coatings was performed for the coatings (GDOES) to analyze the effect of the PSA layer on the formation of PEO coatings (Figure 7 and Figure 8). The evolution of the PSA layer was followed by a sulfur signal change, since PSA is the only source of sulfur in the system (Figure 8). For the PEO layer directly formed on AA2024, the signal of sulfur is on the background level (Figure 7).

From Figure 5 it can be seen that the conversion of the PSA layer to the final PEO coating required a significantly longer time under lower voltages, since the final thickness of the PEO coatings was comparable to the thickness of the original PSA layers. This assumption was supported by the sulfur distribution profiles (Figure 8) through newly formed PEO coatings: There was a clear maximum of the sulfur elemental distribution curve for samples after 30 min of PEO treatment (more evident peak for 350 V, but it was still existing for 400 V). Moreover, the maximums of sulfur distribution profiles were located closer to the outer surface of the PEO coating rather than to the PEO/metallic interface where the PSA was applied originally. For PEO coatings obtained at 350 V for 5 min, the maximum of the sulfur profile was observed at a depth of about 0.5–0.6 μm from the outer surface of PEO coating (with its total thickness of about 1 μm, Figure 8). As can be seen from Figure 1b, the voltage parameters determine the current density of the PEO process. At 450 V, the current density was still quite high (Figure 1b), facilitating the rapid transformation of the PSA layer to PEO. However, under the voltages of 350–400 V, the current density decreased to the minimum values already after 2–5 min of PEO treatment (Figure 1b). This explained why the remains of the PSA layer were still present and sulfur peaks were still visible during GDOES measurements (Figure 8).

According to Figure 8, PEO coatings formed under relatively low voltages (350–400 V) contain a significant amount of sulfur through all the entire PEO coatings which came from the original PSA layer, while under high voltages (450 V), the sulfur signal is practically at the noise level. A significant difference was observed in the ratio between aluminum and oxygen for both the low and high voltage PEO treatments. If aluminum profiles dominated for PEO coatings on bare alloy, for the samples with a previously applied PSA anodization, oxygen profiles significantly increased (Figure 7 and Figure 8).

At the same time, the coatings contained a small amount of phosphorus, the main source of which was the silicate-phosphate electrolyte used in this work. The PSA layer itself, as seen from Figure 8, contains only a small amount of phosphorus, so it cannot be its main source. This is especially noticeable for the PEO coatings formed at a voltage of 350 V, where the amount of phosphorus was much higher (since the conditions for the conversion of the phosphate compounds were not yet sufficiently severe), and its amount smoothly decreased from the outer edge of the coating to the coating/metal interface.

**Figure 9:**
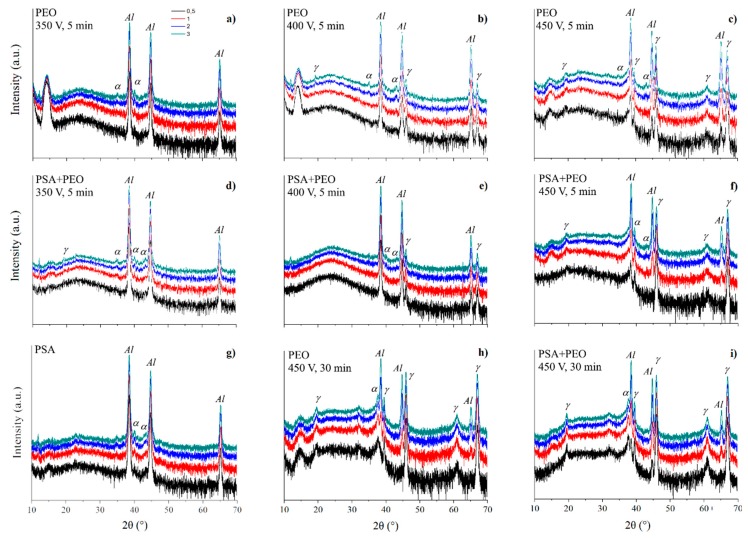
X-ray diffraction (XRD) patterns of PSA (**g**) and PEO coated AA2024 (**a**–**c**,**h**) with pre-treatments PSA (**d**–**f**,**i**) at different voltages (350, 400, and 450 V). Phases of α-Al_2_O_3_ and γ-Al_2_O_3_ denoted by α and γ respectively.

Figure 9 shows the XRD patterns of PSA and PEO coated AA2024 without and with pre-anodizing PSA treatment at different voltages (350–450 V). The formation of PEO coatings in the presence of PSA occured in a manner similar to the case when PSA was absent (the comparison was made using the peaks of α-Al_2_O_3_ at 2θ = 35°, 43°, and peaks of γ-Al_2_O_3_ at 2θ = 39.6°, 45.9°, 67°). However, as can be seen from Figure 9, preliminary anodizing (i.e., PSA in this work) promoted the formation of PEO coatings with lower crystallinity, and even at 450 V, almost no corundum (α-Al_2_O_3_) phase was detected. The characteristic peaks of γ-Al_2_O_3_ also had a lower intensity, and even for the PEO coating formed under 350 V, no peaks of crystalline Al_2_O_3_ were detected. This fact confirmed that the previously formed PSA layer facilitated the sparking initiation, leading to a lower current density during the PEO treatment and reducing the overheating of the system, leading to the formation of more amorphous PEO coatings.

### 3.5. Corrosion Protection Performance of the PEO Coatings

**Figure 10:**
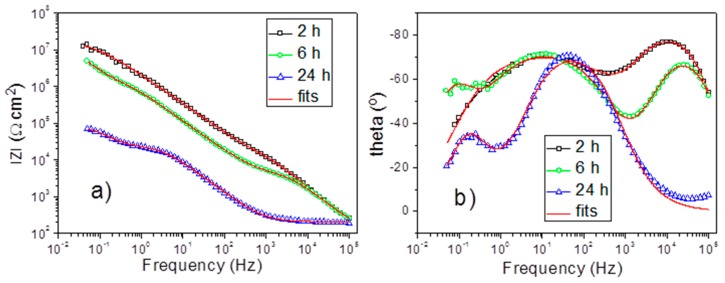
Electrochemical impedance spectroscopy measurements (Bode plots) for PEO-350 V for 15 min, with PSA pre-treatment after immersion in 0.5 wt% NaCl: 2 h, 6 h and 24 h (impedance modulus—**a** and phase angle—**b**).

The corrosion protection performance of the obtained PEO coatings was monitored using the electrochemical impedance spectroscopy (EIS). EIS confers the possibility to quantify important physicochemical parameters of metallic systems during immersion in relevant electrolytes. The evolution of the barrier properties of different coating layers, as well as resistance to the charge transfer, can provide important quantitative insights towards understanding the corrosion resistance of the coated Al alloy substrate. All the systems under study were measured using EIS during 24 h of immersion in NaCl solution. The typical impedance spectra obtained during the evolution of PSA-PEO coated AA2024 in corrosive electrolyte are presented in Figure 10.

**Figure 11:**
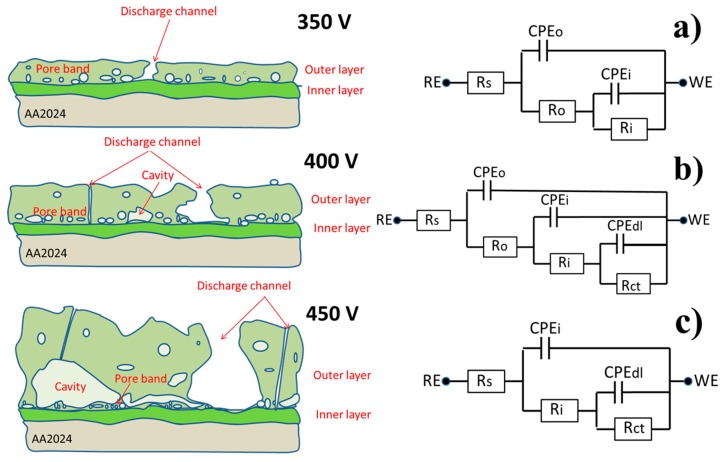
The schematic presentation of the PEO coatings on AA2024, as a function of applied voltage and respective equivalent circuits (**a**) 350V, (**b**) 400V, (**c**) 450V.

At the initial stage of immersion, only two well defined time constants could be evidenced at high (10 kHz) and middle (10 Hz) frequencies, which corresponded to the outer and inner layer of the PEO coating, respectively. After 6 h of immersion, another relaxation process appeared at low frequencies (about 0.1 Hz). This time constant could be ascribed to the starting of electrochemical activities at the metal–electrolyte interface inside the pores. The appearance of a new time constant was accompanied by the simultaneous significant drop of the resistance of the outer layer due to the filling of most of the pores and discharge channels with electrolyte. After 24 h, the pore resistance of the outer layer dropped to the level when it was not distinguishable from the resistance of the surrounding electrolyte anymore. Therefore, the respective high frequency time constant disappeared from the spectra at this stage. The inner layer response and the corrosion-induced low frequency time constant were still well defined on the spectra. Thus, during the evolution of the coated system in the corrosive electrolyte, different equivalent circuits could be used to fit the spectra (Figure 11a–c). The equivalent circuit with three RC (resistance and capacitance) elements enclosed in a cascade like schema (Figure 11b) could be used as a model equivalent to the described electrochemical response of the full system. During the different immersion periods, the simplified equivalent circuits composed of only two time constants could be used. The circuit shown in Figure 11a is applicable to the cases where the corrosion processes had not yet become visible. The circuit without the high frequency time constant (Figure 11c) is adequate for the cases where the high frequency relaxation process is fully hidden by the high electrolyte conductivity in numerous pores of the outer layer.

Figure 10 demonstrates the fitting results of the impedance spectra obtained after 2 h, 6 h, and 24 h, using the equivalent circuits from Figure 11a–c, respectively. In all the cases, the constant phase element was used instead of an ideal capacitor to account for the non-uniformities and associated dispersion of the capacitive response. A high quality of fitting could be evidenced, as also confirmed in Table 1 with acceptable goodness and relatively low errors associated with the main parameters.

**Figure 12:**
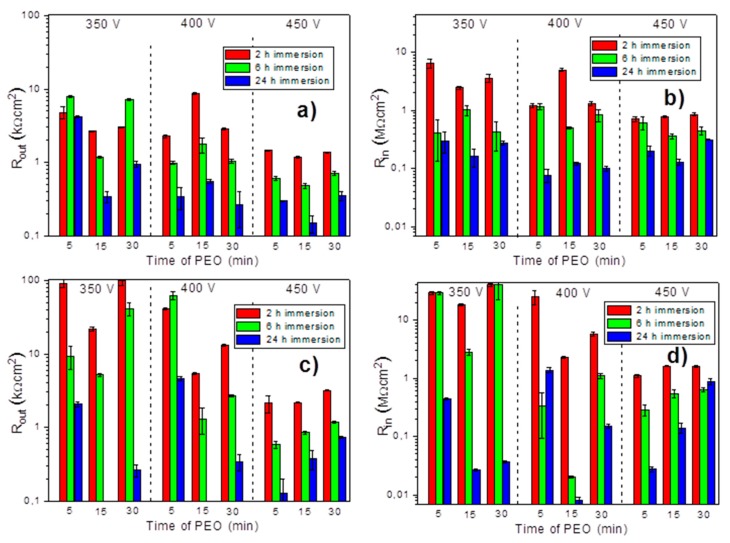
The resistance of the outer (**a**,**c**) and inner (**b**,**d**) layers for the PEO coatings (**a**,**b**) and PEO coatings with pre-anodized PSA layer (**c**,**d**) during immersion 0.5 wt % NaCl (2, 6 and 24 h) (The results of the PEO-PSA sample obtained under 350 V after 24 h of immersion are presented and explained in Figure 10 and Table 1.).

The presented equivalent circuits were systematically applied to fit all the impedance spectra obtained on all the samples. A specific circuit was selected in each case depending on the number of time constants evidenced on the respective spectrum. Figure 12 presents the values of resistance of the outer and inner layer of PEO coatings during the immersion in NaCl solution.

The results clearly demonstrated that higher barrier properties of the outer and inner layers were observed for the systems pre-treated with PSA when the PEO coating was applied at 350 V and 400 V, especially at shorter treatment times. This fact suggested that the PSA layer was not fully destroyed by the applied PEO process under such conditions and it contributed to the corrosion protection of the whole system. In contrast, when the PEO was done at the highest voltage, 450 V, the difference in the initial barrier properties because of PSA pre-treatment became insignificant. The high energy input was sufficient to fully convert the PSA pre-layer without leaving any significant remnants. According to the GDOES and SEM results, it was clear that the pre-deposited PSA layer did not influence the PEO process at 450 V, as well as the structure and composition of the formed coatings. The corrosion resistance of the PSA + PEO coatings, obtained under 450 V, after 24 h of exposure in a corrosive environment did not show any difference to the PEO coatings (without PSA pretreatment) obtained under the same conditions.

The increase of the treatment voltage decreased the initial barrier properties of the outer PEO layer in the case of both types of systems. The higher voltage led to the formation of the thicker layers with more developed porosity. Therefore, the greater thickness of the outer layer had no direct correlation with the barrier properties. It was previously reported in several works that thicker PEO coatings had a sufficiently larger porosity and contained additional cracks, which facilitated the penetration of the corrosive medium to the metal interface [27,28,29,30]. At the same time, the longer duration of the treatment had no obvious effect on this parameter.

### 3.6. Wear Behavior of the Coatings

In this work, the maximal load, under which the coating was still not failing, was determined. A relative study of the abrasion of a steel ball counterpart in contact with PEO coatings was carried out (the static friction partner wear rate (Wk)). The maximum load, the width of the attrition track, and the abrasion diameter of the metal ball were considered.

**Figure 13:**
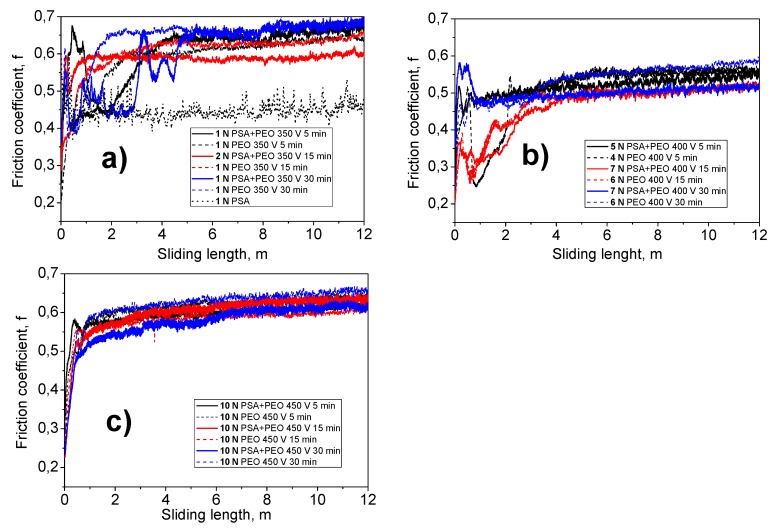
The dependence of the friction coefficient (f) on the abrasion time under a given load of the PSA and the PEO coating, at different voltages (350 V (**a**), 400 V (**b**), and 450 V (**c**)), and times (5–30 min) without and with PSA pre-treatments.

Figure 13 shows the dependence of the friction coefficient (f) on the abrasion time under a given load. It could be seen that under a voltage of 450 V, the most wear-resistant PEO coatings were formed, since they coatings contained a crystalline structure with a mixture of corundum (α-Al_2_O_3_) and γ-Al_2_O_3_. Such coatings withstood a maximum load of 10 N (Table 2 and Table 3), and a change in the friction coefficient throughout the test period was almost identical, regardless of the time of PEO treatment (from 5 to 30 min) and to the preliminary PSA anodization (Figure 13c).

Under the conditions of so-called “soft sparking” (350 V), PEO coatings with an amorphous structure and a thickness of not more than 1 μm were formed. This immediately had an influence on the load bearing capacity of the coatings (Figure 13a). The thickness of such coatings was very small, and the surface roughness (*R_a_*) was approximately 0.25–0.45 ± 0.05 μm. At loads of 1 N, the coatings survived, whilst at loads of 2 N, the coating had already failed. Only the PSA coating failed at 1 N; thus, there was a small improvement due to the PEO treatment at 350 V.

The “sparking” mode (under a voltage of 400 V) could be called a transient mode. With the relatively low thickness of the PEO coating (1.5–2 μm), a significant increase in the coatings wear resistance property was observed. Such coatings were resistant to abrasion under a load of 4–6 N (Table 2 and Table 3). In this case, as can be seen from the distribution of friction coefficient values, the PEO coatings produced on an aluminum alloy with PSA pre-treatment withstood a higher load (5–7 N) than the same coatings formed on a bare alloy (4–6 N).

## 4. Conclusions

Overall, the authors considered the possibility to obtain and optimize the conditions for PEO coating formation on the surface of an aluminum alloy AA2024 after PSA anodizing layer application. To understand the PSA influence, the microstructure and properties were correlated to a direct PEO formation without any anodizing pre-treatment. This work demonstrates that a PEO treatment improves the wear and corrosion resistance to a large extent and offers the option to a localized PEO application for local surface reinforcement. The use of PEO treatment under more stringent oxidation conditions (i.e., high voltage and high current density) has led to the formation of stable crystalline α- and γ-alumina phases, which contribute to the formation of a wear and corrosion resistant layer. The effect of the pre-anodized coating on the conditions and properties of the newly formed PEO coatings, compared with the formed coating with PEO layer formed directly on the bare AA2024 was analyzed. The following conclusions can be reached from this work:

(1) PEO coatings can be formed on preliminary PSA anodized AA2024 alloy, opening the possibility of local reinforcement for future industrial applications.
(2) Preliminary formation of the PSA layer on the surface of aluminum alloy AA2024 facilitates further PEO processes and promotes the formation of PEO-coatings with lower crystallinity.
(3) Under the CC mode, the PSA layer survives under the final voltage of 350 V, whilst at 400 V, there is an intermediate stage; and under 450 V, the PSA layer is fully converted after 5 min of the treatment.
(4) It was shown that during the “sparking” mode (at 400 V) of PEO formation, the PEO coatings formed on the samples with a PSA layer were more wear resistant than the same PEO coatings on bare AA2024.

## Figures and Tables

**Table 1:** Parameters PEO-350 V for 15 min with PSA pre-treatment coating from fitting of the experimental impedance spectra with different equivalent circuits.

Immersion Time (h)	2	6	24
Equivalent Circuit	A	B	C
*R_solut_* (Ω cm^2^)	132.2 ± 6.6	117.3 ± 8.9	206.0 ± 1.7
*R_out_* (kΩ cm^2^)	21.94 ± 1.66	5.197 ± 0.227	-
*Q_out_* (nS cm^−2^)	14.63 ± 1.34	23.07 ± 3.497	-
*n_out_*	0.951 ± 0.008	0.916 ± 0.313	-
*R_in_* (MΩ cm^2^)	18.09 ± 0.80	2.80 ± 0.29	27.05 ± 0.58
*Q_in_* (nS cm^−2^)	95.62 ± 2.47	260.7 ± 10.3	2955.0 ± 920.2
*n_in_*	0.756 ± 0.004	0.813 ± 0.006	0.864 ± 0.004
*R_ct_* (MΩ cm^2^)	-	10.10 ± 1.89	0.053 ± 0.003
*Q_dl_* (μS cm^−2^)	-	0.350 ± 0.024	29300 ± 920
*n_dl_*	-	0.9997 ± 0.0741	1.000 ± 0.029
Goodness	0.534 × 10^−3^	0.518 × 10^−3^	2.484 × 10^−3^

**Table 2:** Parameters wear tests of the PSA and the PEO coating, at different voltages (350–450 V) and times (5–30 min) without and with PSA pre-treatments.

Specimen	Voltage, V	Time, min	Force, N max	Diameter Wear Ball, μm (mean)	Track Width, μm (mean)	Friction Coefficient			Static Friction Partner Wear Rate W_K_, mm^3^ N^−1^ m^−1^
						min	max	average	
PSA	-	-	1 (not)	490.4 ± 61.5	508.8 ± 11.3	0.370	0.600	0.448	7.8773 × 10^−5^
PEO	350	5	1	378.8 ± 12.9	348.8 ± 15.2	0.208	0.661	0.587	2.7867 × 10^−5^
PSA + PEO	350	5	1	412.2 ± 7.8	389.4 ± 9.3	0.240	0.682	0.611	3.9339 × 10^−5^
PEO	350	15	1	397.8 ± 16.4	337.0 ± 12.3	0.242	0.665	0.605	3.3912 × 10^−5^
PSA + PEO	350	15	2	447.8 ± 14.1	435.0 ± 31.4	0.267	0.621	0.586	2.7264 × 10^−5^
PEO	350	30	1	421.0 ± 8.6	378.8 ± 16.4	0.305	0.704	0.642	4.2895 × 10^−5^
PSA + PEO	350	30	1	414.2 ± 4.9	420.6 ± 16.0	0.286	0.702	0.601	4.0108 × 10^−5^
PEO	400	5	4	500.2 ± 21.6	457.8 ± 15.0	0.212	0.575	0.511	2.1354 × 10^−5^
PSA + PEO	400	5	5	535.8 ± 8.7	532.0 ± 23.0	0.236	0.565	0.518	2.2392 × 10^−5^
PEO	400	15	6	485.6 ± 16.6	602.0 ± 31.0	0.189	0.534	0.473	1.2597 × 10^−5^
PSA + PEO	400	15	7	491.4 ± 2.2	459.4 ± 10.7	0.179	0.528	0.462	1.1345 × 10^−5^
PEO	400	30	6	537.2 ± 7.6	524.6 ± 14.5	0.212	0.598	0.534	1.8944 × 10^−5^
PSA + PEO	400	30	7	512.0 ± 2.4	484.4 ± 8.8	0.276	0.582	0.502	1.3417 × 10^−5^
PEO	450	5	10	747.4 ± 6.9	823.2 ± 9.2	0.227	0.653	0.611	4.2676 × 10^−5^
PSA + PEO	450	5	10	700.8 ± 5.4	736.0 ± 9.4	0.278	0.665	0.593	3.2877 × 10^−5^
PEO	450	15	10	708.0 ± 13.9	767.6 ± 8.9	0.248	0.618	0.580	3.4416 × 10^−5^
PSA + PEO	450	15	10	761.4 ± 7.4	851.8 ± 13.3	0.214	0.649	0.600	4.5967 × 10^−5^
PEO	450	30	10	774.4 ± 13.0	855.8 ± 17.0	0.243	0.671	0.620	4.9194 × 10^−5^
PSA + PEO	450	30	10	748.2 ± 7.6	819.0 ± 9.0	0.219	0.630	0.575	4.2898 × 10^−5^

**Table 3:** The photos (optical microscope, 40× magnification) of abrasion tracks on the surface of coatings and steel balls of the PEO coating, at different voltages (350–450 V) and times (5–30 min) without and with PSA pre-treatments, and the PSA and bare AA2024 aluminum alloy.

PSA	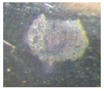	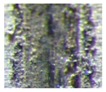	AA2024 bare	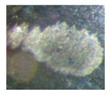		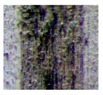
	5 min		15 min		30 min	
PEO 350 V	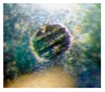	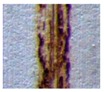	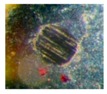	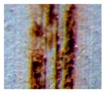	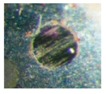	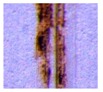
PSA + PEO 350 V	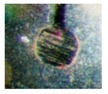	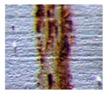	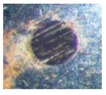	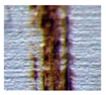	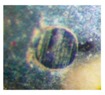	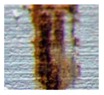
PEO 400 V	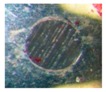	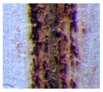	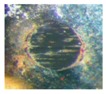	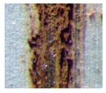	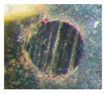	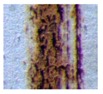
PSA + PEO 400 V	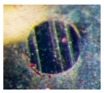	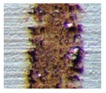	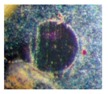	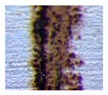	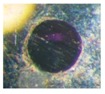	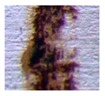
PEO 450 V	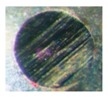	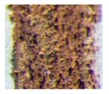	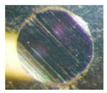	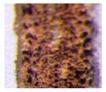	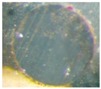	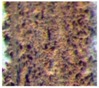
PSA + PEO 450 V	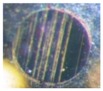	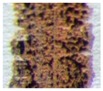	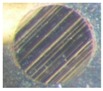	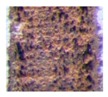	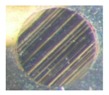	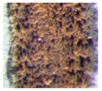
